# Perinatal mental health in medical school curricula: a national scoping survey of British universities and student psychiatry societies

**DOI:** 10.1192/bjb.2022.91

**Published:** 2024-02

**Authors:** Jacob D. King, Grace Crowley, Manal El-Maraghy, William Davis, Archana Jauhari, Charlotte Wilson-Jones

**Affiliations:** 1 Imperial College London, UK; 2 Central and North West London NHS Foundation Trust, UK; 3 South London and Maudsley NHS Foundation Trust, UK; 4 Institute of Psychiatry, Psychology & Neuroscience, King's College London, UK; 5 Essex Partnership University NHS Foundation Trust, Wickford, UK; 6 Anglia Ruskin University, Chelmsford, UK; 7 Lancashire and South Cumbria NHS Foundation Trust, Preston, UK; 8 Powys Teaching Health Board, Welshpool, UK

**Keywords:** Perinatal psychiatry, educational and training, undergraduate curriculum, medical students, medical education

## Abstract

**Aims and method:**

With increasing recognition of the prevalence and impact of perinatal mental health (PMH) disorders comes a responsibility to ensure that tomorrow's doctors can support families during the perinatal period. Online surveys seeking information about the inclusion of PMH education in undergraduate curricula were sent to psychiatry curriculum leads and student psychiatry societies from each university medical school in the UK between April and September 2021.

**Results:**

Responses were received from 32/35 (91.4%) medical schools. Two-thirds reported specific inclusion of PMH content in the core curriculum, typically integrated into general adult psychiatry or obstetric teaching. Students at the remaining schools were all likely to be examined on the topic or see perinatal cases during at least one clinical attachment.

**Clinical implications:**

PMH education offers an opportunity for collaboration between psychiatry and other disciplines. Future work looking at educational case examples with objective outcomes would be valuable.

The fact that perinatal mental health (PMH) disorders are common – affecting up to 20% of women in the perinatal period worldwide – and have significant effects on the life of the mother, the infant, the family and society more broadly is increasingly being recognised.^1–4^ Figures reported by Public Health England estimate that the far-reaching impacts of PMH disorders cost British society between £6.6 and £8.1 billion annually.^5^ Moreover, in the UK suicide remains the most common cause of maternal death between 6 weeks and 1 year postpartum.^6^

Increased awareness of these issues has led to additional government funding for specialist services in the UK.^7^ However, the vast majority of PMH disorders are not identified or treated by specialist psychiatry teams. National Institute of Health and Care Excellence (NICE) guidelines specify the need for referral to secondary care (ideally a specialist PMH psychiatry team) for the treatment of severe mental disorders in the perinatal period, but otherwise recommend referral to the patient's general practitioner (GP) in the first instance,^8^ alongside support from other community services, such as health visitors, social workers and midwives. GPs, obstetricians and gynaecologists, and paediatricians acknowledge the importance of their role in screening and, where appropriate, treating and referring to specialist services for PMH disorders.^9^ Furthermore, primary care and emergency medicine departments see large numbers of women with PMH disorders.^10^ The strong association between predisposing chronic physical health conditions and PMH disorders^11^ also position medical and surgical departments providing long-term care for chronic conditions to identify PMH disorders in their patient populations.

Several sources now call for further work to explore PMH training needs outside of psychiatric services. For example, Noonan and colleagues highlighted the need for more focused consideration of how PMH ‘education strategies and professional development opportunities [can be] appropriately contextualised to the needs of family physicians’.^12^ Seehusen et al found that specific training on postpartum depression had the most significant influence on whether family physicians screened appropriately,^13^ and Leiferman and colleagues reported that a subjective lack of knowledge and training among family physicians was a prominent barrier to screening and treating PMH disorders.^14^ Moreover, several authors have shown that dedicated training of midwives is effective in improving ‘knowledge, skills and attitudes’^15^ as well as, importantly, clinical outcomes relating to support for women with PMH disorders and their families.^16^

A recent UK-based survey of a small sample of Foundation Year 1 doctors reported that although the majority (83.7%) recalled specific teaching on perinatal psychiatry, nearly half (44.9%) had graduated medical school without any self-reported clinical contact with a woman with a PMH disorder.^17^ Given the evidence that even brief training of midwives can lead to improved clinical outcomes, and the current paucity of insight into PMH education at medical schools, it was felt that a scoping survey would be an informative project. This paper describes a national survey to assess the current state of PMH education in UK medical schools. Responses were sought from both medical school psychiatric faculties involved in delivering psychiatry curricula and medical students through their psychiatry societies, to assess both the delivered curriculum and first-hand reports of the education received by students.

## Method

The project team consisted of members of the Faculty of Perinatal Psychiatry at the Royal College of Psychiatrists (RCPsych), psychiatrists experienced in medical education and psychiatry trainees. Two online surveys using the SurveyMonkey platform were developed following a broad review of medical education literature. The surveys were revised after being first tested on pilot participants. One survey was targeted at undergraduate psychiatry curricula leads (or members of the psychiatry faculty involved in curriculum delivery, if no single lead existed) at each UK medical school. The other survey was targeted at members of psychiatry societies from UK medical schools that had a graduating class in 2020. The surveys were identical in content and differed only in the context of their introduction. The RCPsych's Undergraduate Forum – a community of psychiatry curriculum leads invited from each medical school – sent emails with the survey to their members. Similarly, the RCPsych keeps records of registered student psychiatry societies, who were individually contacted by authors and invited to complete the survey. Two UK medical schools did not have student societies registered with the RCPsych at the time of the survey. Each society was asked that one student complete the survey in collaboration with other society members and students of all year groups. Up to four follow-up prompts were sent. Results were collected between April and September 2021, a period during which the UK continued to have restrictions on social gatherings and ‘distance learning’ was common in schools and universities owing to the COVID-19 pandemic: respondents were asked to comment on PMH education ‘currently available’ to students. Data were managed using Stata/IC version 16.1.

Respondents were asked whether there was specific provision for PMH topics in their school's curriculum (yes/no/unsure), whether this was formally assessed (yes/no/unsure) and whether there were student selected components (SSCs) available in PMH topics (yes/no/unsure) (Appendix). SSCs offer students the choice to spend time in one of a range of clinical or academic experiences not normally accessed as part of the mandatory curriculum: they are common to UK medical schools but variably administered and assessed.^18^ Respondents were also asked in what form PMH teaching was delivered and where they believed students at their institutions were likely to be exposed to PMH cases (both from a list of options). Questions provided the opportunity for open free-text comments, which were used to clarify and supplement the findings that emerged from quantitative analysis. We did not perform any separate qualitative analysis on these comments.

For the most part, answers were identical between faculty and student society responses, and minor disagreements were often rationalised with free-text responses. Disagreements between faculty and student responses in relation to delivery of PMH education were handled by synthesising answers in an additive manner, as disagreement was likely to come from PMH topics covered by other parts of the curriculum unbeknown to psychiatry faculty members. Disagreements in relation to where students are likely to be exposed to cases are reported separately, were measured by Cohen's kappa and descriptively compared.

### Ethics approval

Ethics panel approval for this piece of work was deemed not to be necessary by the National Health Service Health Research Authority's self-completed decision tool. The work has been conducted in accordance with the Declaration of Helsinki 1964, and participant responses were managed following the General Data Protection Regulation. Before starting the survey, all participants were required to read a statement advising them that their responses would be kept confidential and may appear anonymised in internal or publicly available external material.

## Results

In total, 24 of 35 (68.6%) curriculum lead surveys were returned, along with 24 of the 33 (72.7%) registered student societies. At least one reply (either faculty, student society or both) was returned by 32 of 35 (91.4%) university medical schools. Both student society and faculty member surveys were returned for 16 (50%) of the replying medical schools. Five respondents answered the survey twice. These duplicate responses were resolved in the following ways: in three cases, responses were identical and the duplicates were therefore removed; in one case, free-text responses rationalised a minor disagreement on the availability of an SSC; and a clarification email was sent in the fifth case. We received 70 free-text responses from 25 (78.1%) responding universities.

### Provision for perinatal mental health topics in the core curriculum

Specific provision for PMH topics was described by 21 (65.6%) medical schools, with 9 reporting that they have no specific provision (28.1%). Two responses from student societies, both without corroborating faculty responses, were unsure.

**Fig. 1:**
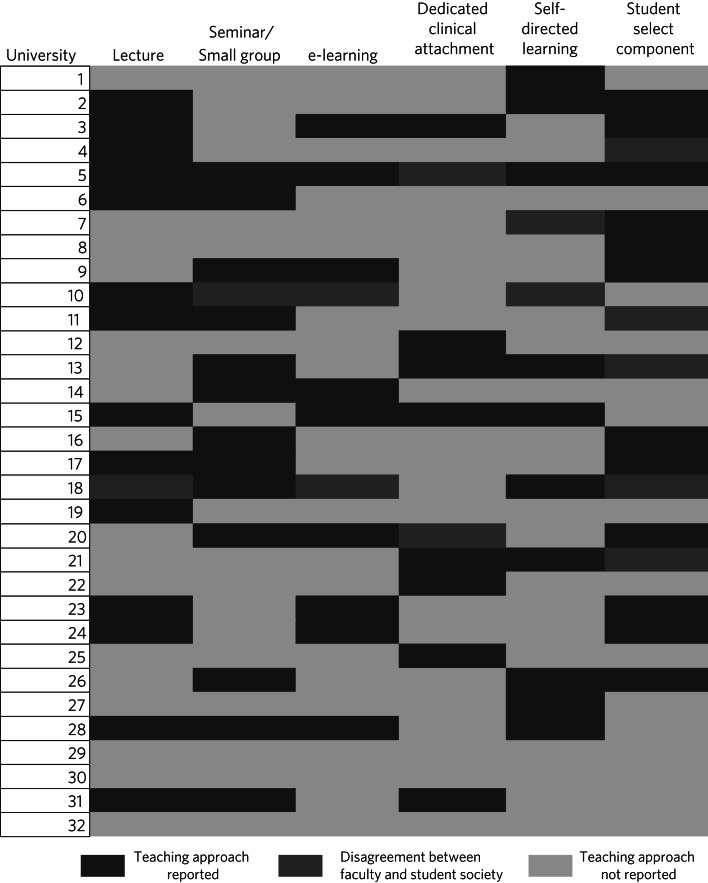
The distribution of reported educational approaches by university.

The format of delivery of PMH topics varied substantially between schools and all but seven universities covering PMH topics reported a multi-modality approach (Fig. 1). Lectures and small group or seminar formats were the most common teaching delivery approaches, used by 15 and 14 universities respectively. A strong sense that PMH topics were included within the delivery of other aspects of the curriculum, rather than specifically designated delivery, emerged from the free-text responses. Respondents suggested that commonly PMH topics are ‘covered here and there in other lectures’ (University 3) and that some schools ‘don't put [PMH topics] as a specific topic in the curriculum but it might be in other parts of the medical school curriculum’ (University 8). Specifically, PMH topics were reported to be ‘discussed within the depression lecture’ (University 28), to ‘occur within lectures on clinical disorders and in tutorial groups’ (University 6) or to be discussed ‘within Psychiatry teaching, Obs & Gynae teaching’ (University 22) or ‘as part of the reproduction block, rather than being a part of the psychiatry block itself’ (University 10).

The modality of delivery of PMH education changed as students progressed through university, with students in the later ‘clinical years’ experiencing PMH topics through clinical teaching during their attachments. Clinical attachments (known internationally as ‘rotations’ or ‘placements’) were the only source of PMH content for 3 (9.4%) universities. Here, coverage came directly through case exposure as well as through case presentation and discussion with senior clinicians, or ‘bedside teaching’ (University 7).

At least 1 respondent from 11 (34.4%) universities reported that PMH topics featured in required e-modules, and 12 (37.5%) universities reported that students were expected to engage in self-directed learning on the topic. Two universities (6.3%) reported that their only exposure to PMH topics was through self-directed learning.

### Exposure to perinatal mental health cases

**Fig. 2:**
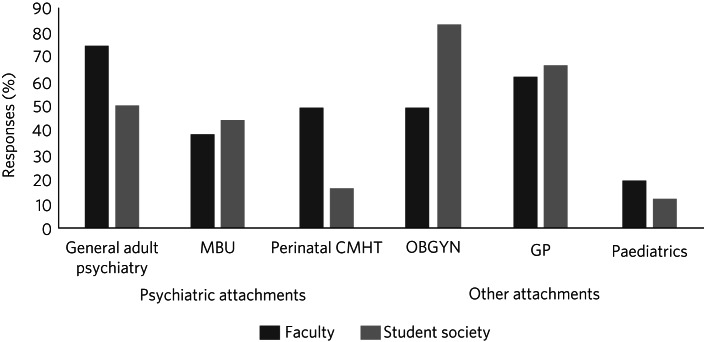
Medical students’ exposure to perinatal mental health cases during medical training, as reported by faculty members and students. MBU, mother and baby unit; CMHT, community mental health team; OBGYN, obstetrics and gynaecology; GP, general practice.

Respondents were asked during which clinical attachments they believed students at their institutions were likely to encounter women with PMH disorders. All respondents suggested that students were likely to see PMH cases in at least one attachment at their medical school. The most common settings reported by faculty members were general adult psychiatry attachments (18/24; 75.0%) and GP attachments (15/24; 62.5%). Among students however, obstetrics and gynaecology (20/24; 83.3%) and GP attachments (16/24; 66.7%) were assessed as most likely, with general adult psychiatry highlighted by 12/24 (50%) of student societies (Fig. 2). Where responses were received from both faculty and student societies, there was poor agreement as assessed by Cohen's kappa (κ = 0.33). No ‘other’ options were suggested.

Attachments on in-patient perinatal mother and baby units (MBUs) were available to students at 14 of the 32 universities (43.8%). Free-text comments identified only 3 universities (9.4%) where attachments on an MBU are facilitated for all, for example with ‘very brief (half day) clinical attachment on mother/baby unit’ (University 12). For most universities, MBU attachments did not represent a routine attachment, but rather there was opportunity for students to undertake an SSC or arrange their own experience *ad hoc*.

Attachments with perinatal community mental health teams (PCMHTs) were subject to the most disagreement between faculty and student society respondents. While 40.6% (13/32) of faculty members reported some provision for a PCMHT attachment, only 3 student societies confirmed that students were likely to see patients there. However, two respondents highlighted the impact of the pandemic on these attachments: ‘COVID made [attachments] more complex [to arrange] and the team seem to have lost their place in perinatal clinic’ (University 15). Another respondent commented that their ‘perinatal CMHT is just being started up, so this will be another source of exposure’ (University 10).

### Assessment of perinatal mental health topics

Twenty-one universities (65.6%) described the potential for at least some assessment of all students on PMH topics, 7 (21.9%) reported that there was no facilitation of assessment in this field and 4 (12.5%) were unclear. Of those with some assessment capacity, all provided further clarification with open-text responses. Some universities suggested that PMH topics were always assessed, usually as part of written examinations in either single best answer or multiple choice question formats as part of an end-of-block assessment; others explained that questions on PMH topics were included in a larger bank of questions that could come up in written examinations but were not guaranteed to appear. Fourteen universities (43.8%) suggested that PMH topics could feature in objective structured clinical examinations (OSCEs), but most implied that although possible, this was rarely, if ever, the case: ‘I'm not aware of any previous OSCE stations that have been based on perinatal mental health; however, this definitely could come up as part of a summative assessment’ (University 10).

### Student selected components

Twelve of 32 (37.5%) responding universities reported provision for a dedicated PMH SSC, 15 (46.9%) suggested these were not available, and 5 (15.6%) were unsure. Three of those facilitating SSCs (25%) reported that students would not ordinarily be offered PMH SSCs, but that students could themselves arrange them on an *ad hoc* basis if they wished. Based on free-text responses, the most widely available PMH SSCs were attachment to MBUs, which in some cases are not otherwise accessible in the mandatory curriculum.

Universities hosting a PMH SSC reported typically being able to facilitate these for 1–2 students annually. The exceptions were one university which suggested a theoretical annual capacity for 16 students across year groups, and another which reported offering several different PMH SSCs, each within a different perinatal clinical service or the linked academic department, which combined could theoretically accommodate ‘1–30 students’ (University 9) each year. Three universities (9.4%) suggested that academic PMH research groups have currently or historically offered SSCs placed in their research units.

## Discussion

To our knowledge, this is the first time that UK medical school faculties and student societies have been surveyed about PMH content in their curricula. Although responses suggested that around one-quarter of medical schools have no specific provision for PMH topics in their curriculum, all of these universities also suggested that students are either expected to engage in self-directed learning, potentially examined on the topic or will likely come across cases during at least one of their clinical attachments.

Experiences in specialist PMH services or SSCs were available at only a minority of medical schools and were usually only available on an *ad hoc* basis rather than for all students. Given the recent increase in UK government funding, and along with it the formation of several new PCMHTs and MBUs,^7^ the capacity to host medical students may increase as these specialist teams become more established.

These results also demonstrated that a proportion of PMH education is delivered by other departments, particularly obstetrics and gynaecology. A discrepancy between faculty and student society reporting on where students were likely to encounter clinical cases might also highlight the psychiatry faculty members’ lack of awareness of the PMH experiences students are receiving elsewhere and might imply limited formal interdisciplinary education.

With time pressures in the undergraduate medical curriculum, more of any topic must be settled against the content it displaces, and there are many difficulties which come with updating and implementing a medical curriculum in line with national clinical priorities.^19^ For example, although Scotland has in place a PMH training curriculum for health education,^20^ in our results Scottish medical schools were comparable to overall figures, with specific provision reported by 3/5 (60%), no provision by one and one ‘not sure’ response. Free-text comments from these medical schools did not refer to the national PMH training curriculum.

In the UK, both the Royal College of Psychiatrists and the Royal College of Obstetricians and Gynaecologists specify PMH content in their national guidance for the undergraduate curriculum, the latter highlighting PMH as a core condition during pregnancy for educational focus.^21,22^ There are significant collaborative opportunities for psychiatrists and obstetricians and gynaecologists to work together to deliver PMH education to medical students, especially (as our results suggest) since both are already providing this.

### Limitations and future directions

Although a strong response rate has been beneficial to this survey, receiving more pairs of faculty and student society responses might have given a more representative account of the delivered and received PMH content in each medical school, especially as the discrepancies here have been illuminating. It follows that a crucial limitation is the absence of perspectives of other curriculum leads, particularly from OB/GYN and general practice faculty members. Moreover, as participants were not asked to consent to publishing their identifiable responses we were unable to report further breakdowns of responses. This novel scoping survey, containing only six questions, was limited in other ways and future work ought look at the delivery of the full curriculum for perinatal mental health topics, including the informal curriculum, estimates of total time allocated to perinatal topics, whether specific aspects of PMH were differentially delivered by the different specialties, and why schools chose to deliver the content in the way that they have. Future work focusing on undergraduate PMH education could look to collect case studies of interdisciplinary education and measure objective educational outcomes of perinatal education interventions among medical students.

Survey items were piloted on trial participants and developed from reviews of similar surveys. However, they are novel items and have not be validated, independently reviewed or used previously, which may limit their validity. Furthermore, the reports of student societies, while representing the student body, are unlikely to be able to capture the experiences of all students and therefore are only a proxy for overall student experience. We suspect that some responses, particularly from student societies concerning perinatal attachments, were affected by recent attachment changes owing to the COVID pandemic, and therefore the results of this survey may not accurately represent pre- or post-pandemic arrangements.

### Implications

There is a strong clinical, financial and moral case for undergraduate education in perinatal mental health. Our finding that psychiatry faculty members may underestimate the opportunities for PMH case exposure outside of the psychiatry curriculum reveals an attractive opportunity to improve collaboration between medical specialties, particularly as the integration of perinatal psychiatry and obstetrics and gynaecology is recommended in the Royal College of Psychiatrists’ advice to medical schools.^22^ This could be taken further, to include nursing and midwifery colleagues in PCMHTs and antenatal clinics, which may be more accessible and generalisable for student attachments than, for example, MBUs. The recent advances in online platforms and distance learning models could furthermore facilitate this. A commitment from medical schools for PMH topics to be taught in undergraduate medical curricula is likely to improve the competency of graduates in this increasingly important field.

## Data Availability

Raw data are available on request from the corresponding author.

## About the authors

**Jacob D. King** is a National Institute for Health and Care Research (NIHR) Academic Clinical Fellow in the Division of Psychiatry, Imperial College London, UK, and Central and North West London NHS Foundation Trust, UK. **Grace Crowley** is an NIHR Academic Clinical Fellow in South London and Maudsley NHS Foundation Trust, UK, and the Institute of Psychiatry, Psychology & Neuroscience, King's College London, UK. **Manal El-Maraghy** is a consultant perinatal psychiatrist with Essex Partnership University NHS Foundation Trust, Wickford, UK, and a visiting clinical senior lecturer at Anglia Ruskin University, Chelmsford, UK. **William Davis** is a speciality registrar in general adult psychiatry with Lancashire and South Cumbria NHS Foundation Trust, Preston, UK. **Archana Jauhari** is a consultant perinatal psychiatrist with Powys Teaching Health Board, Welshpool, UK. **Charlotte Wilson-Jones** is Director of Mental Health Education at the Institute of Psychiatry, Psychology & Neuroscience, King's College London, UK.

## Appendix

Summary of survey items

1. On behalf of which medical school are you completing this survey? [Free text response]
2. Is there specific provision for perinatal mental health topics in your institution's compulsory psychiatry curriculum? [Yes/No/Not sure]
3. If yes, what form does the content delivery take? Please tick all that apply. [Single lecture; Lecture series; Seminars/small group discussion; Clinical attachment; Self-directed learning; e-learning/online modules; Other (free text); There is no provision for perinatal mental health topics]
4. Where are students at your medical school likely to be exposed to perinatal mental health cases? Please tick all that apply. [General adult psychiatry attachments; Perinatal community mental health teams; Mother and baby units (MBUs); Obstetrics attachments (OB/GYN); General practice (GP) attachments; Paediatrics attachments; Public health attachments; Other (Free text)]
5. Is an understanding of perinatal mental health topics assessed as part of your medical school's assessment of psychiatric knowledge and competence? And if so, what form does this take? (for example, examination, practical competencies – summative or formative?) [Yes/No/Not sure; Free text reply]
6. Does your institution offer a dedicated student selected component (SSC) in perinatal mental health? If yes, in what service are the attachments facilitated and how many students each year can be accommodated? [Yes/No/Not sure; Free text reply]
